# Exploring the Experiences of People with Obesity Using Portion Control Tools—A Qualitative Study

**DOI:** 10.3390/nu11051095

**Published:** 2019-05-17

**Authors:** Eva Almiron-Roig, Anne Majumdar, David Vaughan, Susan A. Jebb

**Affiliations:** 1 MRC Elsie Widdowson Laboratory, Cambridge CB1 9NL, UK; susan.jebb@phc.ox.ac.uk; 2 Centre for Nutrition Research, University of Navarra, 31008 Pamplona, Spain; 3 Navarra Institute for Health Research (IdiSNa), 31008 Pamplona, Spain; 4 St Mary’s University, London TW1 4SX, UK; anne.majumdar@stmarys.ac.uk; 5 Betsi Cadwaladr University Health Board, Bangor LL57 2PW, UK; aceii.sbhsf@gmail.com; 6 Nuffield Department of Primary Care Health Sciences, University of Oxford, Oxford OX2 6GG, UK

**Keywords:** portion size, education, awareness, portion-control tool, calibrated tableware

## Abstract

Large portion sizes increase consumption and eating smaller portions is recommended as a weight control strategy. However, many people report difficulties enacting this advice. This study examined the experience of individuals using two commercially available portion-control tools to try to manage their weight. In a crossover design, 29 adults with obesity (18 women) who had attended a previous weight loss intervention in the community were invited to use two portion-control tool sets over a period of four weeks (two weeks each) and to complete a semi-structured questionnaire about their experience. The tools were a guided crockery set (sector plate, calibrated bowl, and calibrated glass) and a set of calibrated serving spoons (one for starch, one for protein, and one for vegetables). Data were analyzed using thematic framework analysis. A key theme was related to the educational benefits of the tools, such as generating awareness, guidance, and gaining an independent ability to judge appropriate portions. Other key themes were tool usability, acceptability, and feasibility of usage. Barriers identified by participants included unclear markings/instructions and the inconvenience of using the tool when eating away from home. Overall, the tools were perceived to be educationally useful, easy to use, and potentially effective for learning to control portions, which suggested that these instruments could help in weight management interventions alongside other strategies. Elements of the tool design could influence the ability of participants to adhere to using the tool, and hence allow the educational effect to be mediated.

## 1. Introduction

Evidence suggests that exposure to large portions consistently leads to increased intakes and may increase the risk for obesity and related diseases [1,2]. Portion control of high energy density foods is frequently recommended as a key element for weight management [3], however, very little is known about how to help people eat portion-controlled meals. Educating people on the effects of large portions [4] or using portion-size information on food labels [5] has had only a limited effect. This may be because choosing large portions can occur without awareness for some people [6,7], facilitated by cultural norms making us think that large portions are the “norm” [8], and by price incentives to consume larger portions [9], amongst other factors. Enabling people to adjust behavior towards healthier food portions is a real challenge. While greater changes in our food environment may be needed to impact on portion control at the population level, some individual-level strategies have shown promise, including the use of portion-controlled meals, reduced pack sizes, use of modified tableware, attentive eating, and portion-control strategies as part of weight management programs [10]. Traditionally, educational aids to guide portion sizes have been used as part of such programs, however, most of these aids are image- or text-based [11,12,13] and deemed to be of limited effect [14,15], in part due to inconsistent portion size standards [16]. Recently, three-dimensional portion-control tools have been commercialized, with claims to control portion sizes by either physically delineating volume (e.g., portion pots [17] and guided tableware [18]) or by including visual prompts for appropriate amounts, such as calibration marks in tableware and serving utensils [18,19]. Such instruments, being practical in nature, have the potential to be a useful strategy that enables individuals to cope with a food and drink environment where large portions are often the norm [1,9]. However, the exact mechanisms by which such tools may control portion size or help in weight management are not fully understood. Several recent systematic reviews [1,2,20] confirm that people serve themselves larger food portions when using larger tableware or containers, and that the shape, depth, and capacity of the container or serving utensils may modulate the way we perceive a portion and how much we consume. Specific tools to guide portion size may also provide a visual reference point for the amount of food to be consumed [6,21,22]. Tools containing images as well as those including three-dimensional stimuli (e.g., volume in measuring utensils) may increase the user’s attention and facilitate learning versus other types of tools [23,24,25]. 

However, evidence of the effectiveness of instruments featuring these attributes is limited [21,26,27]. A 12-month randomized controlled trial [15] testing a combined portion-control tool set which included a food scale, measuring cups and spoons, and two image-based portion size guides, showed no effect of the portion-control tool set versus standard advice, however, both groups followed a rigorous lifestyle modification program which may have attenuated differences. In addition, the particular impact of calibrated tools designed for eating, as opposed to measuring, was not explored. Previous studies using calibrated plates have shown these tools to have potential usefulness as additional weight management strategies [21,26,27]. However, such studies did not explore the consumer experience of the tools, that is to say, whether they lead to better understanding of appropriate portions, what barriers participants experience when using the tools, or the acceptability and perceived functionality of the instrument (i.e., whether the tool was seen as effective to control portions). These aspects can impact usage and adherence, affecting the potential effectiveness of tools, and therefore investigating the experiences of users is key before application at a large scale. As part of a pilot study, we have previously reported the acceptability and potential effectiveness of two different portion-control tools (a guided crockery set including plate, bowl, and glass; and a set of calibrated serving spoons for starch, vegetable, and protein) amongst patients with obesity, using a quantitative questionnaire [22]. Here, we report the direct experiences of the participants using these instruments in a qualitative analysis from the same study, using an inductive approach to explore the perceptions of the participants in more depth and to inform future development and usage of these tools.

## 2. Materials and Methods 

Adult volunteers were recruited from the Lifestyle and Weight Management Services (LWMS) at the Wirral Community NHS Foundation Trust in Birkenhead and surrounding area between November 2013 and August 2014. This community is located within the Borough of Wirral and is one of the most deprived regions in England [28]. A sample of 43 patients attending a public weight loss service who had recently completed 7–12 weeks of a community weight loss program (BMI > 35 kg/m^2^ at the start of the program), were invited to try at home two portion-control tool sets, each for two weeks, in random order. Sample size for this exploratory qualitative analysis was based on sample requirements for the quantitative section of the project (30 completers) and considered likely to reach saturation for this study [29]. Participants were asked to complete a questionnaire containing open-ended questions to generate comments (while they were using each set of tools) and body weight was recorded before and after the completion of the study. The study protocol was approved by the West Midlands NHS Health Research Authority ethics committee (reference number 12/WM/0426). All participants provided a written informed consent and those completing the study were offered the opportunity to keep one of the portion-control tool sets (of their choice) as a token of appreciation for their time. All personal information was kept confidential and retained exclusively at the LWMS as part of the clinical notes for each patient. 

### 2.1. Portion-Control Tools

**Figure 1:**
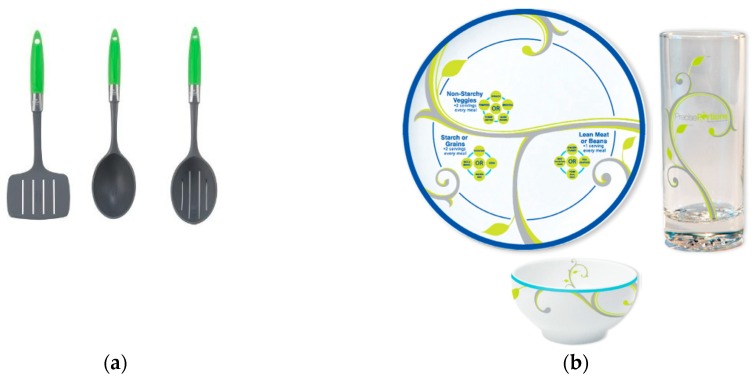
Portion-control tools used to explore the experiences of 29 individuals with obesity trying to manage their weight. (**a**) The set of plastic serving utensils included a serving spoon with capacity for 1 portion of starch (0.5 cup), a serving spoon for 1 portion of vegetables (0.5 cup cooked, 1 cup raw), and a serving spatula for 1 portion of protein (2.5 oz.), based on the USDA standards (source: HealthySteps™ [19]). (**b**) The crockery set included a 9-inch plate decorated with leaves depicting 3 sectors and labels to indicate food types (non-starchy vegetables, protein, and starch) in each sector; a crockery bowl with disguised marks for 0.5 cup, 1 cup, 1.5 cup; and a clear, tall glass with disguised marks for 4 oz. and 8 oz. (source: Precise Portions Nutrition Learning System [18]).

The portion-control tool sets were a set of serving utensils and a set of crockery tableware (Figure 1). These tools were selected based on the results of a previous literature and expert consultation, as well as from an anonymous survey amongst 66 individuals with obesity (BMI > 35 kg/m^2^) attending weight management services in the same community [22]. The consultation process suggested that for portion-control tools to be effective they need to be versatile across a range of foods, culturally acceptable, easy to use, resistant to wear and tear, and correspond with reference portion sizes. These criteria were used to design an anonymous poll where participants ranked four portion-control tool sets meeting the above criteria, in order of preference, after receiving a short demonstration of their use. The four tool sets included an all-in-one portion pot [30], a color-coded measuring portion pot and a measuring spoon set [17,31], plus the crockery and the serving spoons sets (Figure 1) [18,19], which were the two tool sets which were ranked highest. For the present study, both tools were accompanied by a simple set of instructions based on the manufacturer’s information including details of the components of each tool set, correspondence to reference portion sizes, and information on heat resistance and cleaning instructions. Each participant was asked to use each of the tools for a 2-week period, in random order. Participants saw their lifestyle advisor at the start, middle and end of the study period as part of their routine health care visits. To avoid confounding, participants who took part in the initial anonymous poll (*n* = 66) were excluded from the trial.

### 2.2. Data Collection

Qualitative data was collected by questionnaire including five open-ended questions designed to elicit the experiences of the participants while using the tool, including what they liked and disliked, where it had proved useful or less useful, and factors that encouraged or prevented use of the tool (Supplementary Material File S1, questions 5, 7, 9, 10 and spontaneous comments space). Given that feelings and perceptions around the portion-control tools were likely to arise while using the tool at home, an open questionnaire to be completed in real time was considered appropriate to minimize the need for participants to recall past experiences and relate these to a specific tool. The prepiloting of the questionnaire took place amongst 7 independent participants with obesity from the same geographical area before implementation.

Participants met with the lifestyle advisor during their first study visit and were informed about the tools and how to use them. They were given the questionnaires to complete, together with the first portion-control tool set and instructions for use. Participants were asked to complete the questionnaire while using the tools at home, and to return the completed questionnaire and portion-control tools at their second visit two weeks later. During visit two, participants met with the lifestyle advisor again to review their experience, returned the materials, and received the second tool set with instructions plus a second copy of the questionnaire, which they completed as per the first visit. This questionnaire and the second tool set were returned two weeks later (end of the study). During each visit, staff were offered the opportunity to record, in writing, any spontaneous comments from participants in the participant’s case report form (open box stating “include any comments made by volunteers during the study visit”).

### 2.3. Data Analysis

The inductive approach was used for analysis [32]. Thematic framework analysis was used to identify themes both from a set of predefined research questions and from the responses of the participants [33,34]. Coding was completed by two independent trained investigators (authors AM and EAR, not involved in the data collection step). Two coders were employed given their backgrounds (research dietitians) in order to avoid biases and expectations to influence identification of themes. In addition, initial themes were verified by an external qualitative analysis expert, not involved in the study, with a background in public health. Disagreements were discussed to reach the final consensus. Data was coded and analyzed following the five stages of thematic framework analysis, which are: (a) familiarization with the data; (b) identification of a set of thematic framework codes; (c) systematic application of the thematic framework to all transcripts (indexing); (d) creation of a chart (matrix) for each theme by case and code; and (e) mapping and interpretation, i.e., analyzing themes and their interrelation, taking into account the original research questions and concepts generated inductively from the data [35,36]. A constructivist approach was used with the researchers forming a co-construction between themselves and the data. Illustrative extracts are presented for each theme. Further details are available on request.

## 3. Results

### 3.1. Participants

Participant characteristics are shown in Table 1. The sample consisted of adult men and women with obesity with a mean (range) BMI of 40.9 (30.8–54.7) kg/m^2^. Most participants were of White ethnic background, with no experience of portion-size tools and who normally prepared meals for themselves and others. About half were trying to follow a healthy eating plan or a reduced carbohydrate diet. Participants lost on average 1.7 ± 4.1 kg over the four-week intervention period. Retention at the time of the final follow up was 70% (30/43). After excluding one invalid questionnaire (wrong tool code recorded), results for 29 participants (11 men and 18 women) were retained for analyses. Fourteen participants used the crockery set first and 15 used the serving spoon set first. The number of respondents was similar across tool sets and the proportion of men vs. women responding to each question (average 39 ± 9% men) was similar across tools and similar to the overall sample (38% men). There were no recorded comments from staff.

### 3.2. Main Themes 

Across tools and questions, charting and interpretation of each aspect of the responses identified by the thematic framework, led to the identification of a range of experiences which were captured in five identified over-arching themes (Table 2). The dominant theme was the *educational value* of the tool, which included main themes such as increased awareness of previously oversized portions, confirmation of portion sizes, and developing ability to appropriately portion food independently. Secondly, *usability* with themes included aspects of functionality, versatility, and ease of use. Third, *acceptability*, including quality and design of the tool. Fourthly, *feasibility* covering convenience, appropriateness, and adherence. Fifth, *overall experience* with the tool. 

#### 3.2.1. Educational 

This theme emerged repeatedly for both tools in equal prevalence and related to gaining knowledge about portion sizes from using the tools (i.e., allowing to learn about appropriate portion sizes for their needs, such as what do they look like for each type of food group, and becoming aware of the difference between habitual portions and recommended ones). The tools were reported to have made participants aware of previously oversized portions, where they most commonly had excessive carbohydrate and protein portions and an insufficient vegetable portion.

“*It is so easy to put too much on the plate without realizing it*”, (ID119, 53 years old, F, 40.3 kg/m^2^, lost 4.4 kg).

“*Prior to use, I tended to over eat pasta, rice etc. and under eat vegetables*”, (ID140, 24 years old, F, 35.4 kg/m^2^, lost 2.4 kg).

Participants also felt that the tools were a useful visual aid to compare portions against. Some participants felt that they had strived to keep portions to size previously but that they lacked confidence, and therefore the tools were helpful to boost or confirm their knowledge. 

“*The portion markings on the plate took away the guesswork of whether you had the correct amounts of each group*”, (ID114, 58 years old, M, 43.9 kg/m^2^, no weight lost).

Most participants reported that they were able to achieve improved portion control, and they felt that when they were using the tools it was easier to eat less and feel fuller sooner, plus, for the serving spoons, being less likely to “cheat” on portions. The tools were reported to facilitate reduced food intake, feelings of fullness, and ability to serve a balanced meal (i.e., consuming the appropriate amount of starch, protein, and vegetables relative to each other). 

“*The carbs and veg [spoons] were the most useful to make sure that my meal was balanced for example, making sure that my meal was big enough to fill me while keeping my protein and carbs at the correct size while bulking up with veg*”, (ID106, 31 years old, M, 54.4 kg/m^2^, lost 6.2 kg).

There was a clear learning element to the tool, whereby participants reported that they were subsequently able to put in place the educational guidance of the tool and achieve correct portion sizes independently. 

“*Towards end of trial period, [I] started getting to know portions without use of tools, but still checked to make sure*”, (ID114, 58 years old, M, 43.9 kg/m^2^, no weight lost).

“*Useful for sizing but not needed once I got used to quantities*”, (ID125, 63 years old, F, 41.7 kg/m^2^, lost 1.7 kg).

Some participants reported having reduced the size of their usual tableware as a result of using the crockery tool, and that they adjusted to the new quantities after using the crockery or the spoons. 

#### 3.2.2. Usability 

This theme included the sub-themes ease of use, functionality, and versatility of the tool to be utilized in meal preparation and to manage portion sizes for weight loss. Ease of use was mainly associated with design elements. For the serving spoons, ease of use related to being “hassle-free” and simple. For the crockery, usability related to the clarity of marks or divisions on the plate, ease in measuring or understanding, convenience, and practicality. In contrast, one participant found the marks unclear, especially on the bowl and glass. 

Versatility was an emergent theme that impacted on the functionality of the tools for portion control. Not knowing how to use either tool for mixed food (e.g., salads, rice, and pasta dishes), or dry food (uncooked grain/pasta) led to difficulty in using both sets. Low versatility of the tool with respect to particular users and contexts was also identified as a barrier to use. Some of these sub-themes emerged under the functionality theme indicating that lack of versatility affected perceived functionality (e.g., for correct vegetable intake, spreads, breakfast cereal, savory snacks and beans). 

“*It would have been better to have tool to measure ‘raw’ carbs to save waste*”, (ID102, 45 years old, F, 49.5 kg/m^2^, gained 2 kg). 

*“Kids were still hungry. Only used for myself”*, (ID105, 40 years old, M, 36.6 kg/m^2^, gained 0.8 kg).

Requiring time to measure out food or for getting used to the tool were also associated with difficulty in use. When eating with others, some participants felt extra crockery for a dinner partner would increase usability. In terms of functionality, participants judged the tools to be useful for what they were designed to do if they helped with portion size judgements, for example, through the presence of marks or divisions which improved the precision of servings, or via the container itself (e.g., restricting on how much to serve). For the crockery, this related particularly to the tool allowing the visual comparison of usual portions against recommended, restricting or limiting amounts, plus allowing direct measurement of some foods (e.g., bowl for cereal). The plate was the crockery component most frequently cited as useful. For the serving spoons, functionality was linked with simple yet accurate and precise measuring (with no need for weighing). The starch serving spoon was the component found most useful, followed by the vegetable spoon. 

“*The carbs and veg [spoons] were the most useful to make sure that my meal was balanced […]*”, (ID106, 31 years old, M, 54.4 kg/m^2^, lost 6.2 kg).

For some, the crockery was not perceived as useful and sometimes unsuitable for certain foods (e.g., the plate for mixed dishes and the glass for tea, coffee or water), or unsuitable to specific users like children. Some participants indicated negative aspects relating to functionality due to the failure to achieve a portion goal, linked with remaining hungry (implying too small portions) and consequently snacking more, having second helpings, or as in the case of the crockery, the possibility of piling food upwards. 

[Referring to the plate], “*although the vegetable section was too small in my opinion! I like to fill up on vegetables and felt hungrier and snacked more*”, (ID102, 45 years old, F, 49.5 kg/m^2^, gained 2 kg). 

“*Although the plate is well marked, it would not stop one piling upwards*”, (ID116, 72 years old, M, 39.2 kg/m^2^, lost 1 kg).

#### 3.2.3. Acceptability 

Acceptability emerged as a theme that appeared to strongly impact on adherence to using the tool plus compliance of usage to achieve portion control, and encompassed quality and tool attractiveness. Perceiving the tools as being of low quality was associated with a negative experience and related mostly to disliking the weight, material, and finish. Quality issues was a stronger theme for the spoons than for the crockery.

On the other hand, the tool being desirable in pattern, color, material, size, weight, and general design were themes associated with acceptance for both tool sets. Personal preferences for the color and size of the tools affected perceptions of the tools on an individual basis.

“*The crockery plate is perfect size so is the bowl. Didn’t find much use for the glass, had plenty of the same size in the home*”, (ID150, 40 years old, M, unavailable BMI, lost 0.3 kg).

#### 3.2.4. Feasibility 

This theme included sub-themes relating to adherence, appropriateness, and convenience. Poor adherence leading to inconsistent use of the tools was associated with forgetting to use the tools, using them as a theoretical guide rather than for actual eating or serving, and using only certain components. Forgetting to use the tool was a common sub-theme, often linked to someone else cooking or serving the meal, or forgetting to use them for specific foods or erratic use (e.g., discontinuation during festivities). The latter was linked to appropriateness, including eating with guests, eating on the go, at work, or with children. 

“*I would of liked to use the plate for my lunch. However taking it to work was inconvenient but I did use it to measure the portions*”, (ID140, 24 years old, F, 35.4 kg/m^2^, lost 2.4 kg).

[Referring to the spoons] “*The tools were given too close to Xmas. I was away for Xmas and had family for the New Year so never used them […]*”, (ID105, 40 years old, M, 36.6 kg/m^2^, gained 0.8 kg).

#### 3.2.5. Overall experience 

Many participants were extremely positive towards the tools and expressed appreciation for receiving them. 

“*Very happy to use them. Right size plate and bowl—convenient and practical, pleasant pattern*”, (ID144, 67 years old, F, 35.5 kg/m^2^, lost 0.1 kg).

The spoons were perceived to have added value, with wider uses in the household including use in cooking, serving others at the table, helping others with portion control, and reducing time plus waste.

“*It helped considerably when portioning/serving food not only for myself but also my family, also helping to reduce […] food wastage*”, (ID142, 57 years old, F, 44.3 kg/m^2^, missing data for weight change).

Sub-themes included wishing to own the tool and using it consistently, having enjoyed the current design, for it to be commercially available and reasonably priced, desire to buy it for self and others, preferring the tools over their own utensils, perceiving them as a positive thing, and planning to use them with more foods.

Improvements suggested for the crockery set referred to marks/divisions or instructions, vegetables division, size, weight, and color.

“*When having a pasta dish I thought it would be good if there was a clear guide of how much to serve*”, (ID102, 45 years old, F, 49.5 kg/m^2^, gained 2 kg). 

For the serving spoons, improvements related to the quality, versatility, and instructions for use across foods. However, being happy with the current design was the most prevalent theme and frequently related to functionality.

“*The tools are perfect for what they are designed for*”, (ID119, 53 years old, F, 40.3 kg/m^2^, lost 4.4 kg).

“*Maybe include some of the lesser known uses for products for example, include cereals, crisps and beans. Other than that the product is perfect for helping eat the correct portions, it’s all about making sure that I stick to the recommended portions*”, (ID106, 31 years old, M, 54.4 kg/m^2^, lost 6.2 kg).

Further suggestions included combining the tools, making them suitable for other family members, and making them accessible, suggesting an overall positive experience.

## 4. Discussion

This study examined the experience of a group of adults with obesity using eating and serving utensils specifically designed to improve portion control in the home environment. Overall, participants found both sets equally acceptable, easy to use, and educationally useful. Most of the participants who reported finding the tool(s) or part of them helpful had an initial BMI > 40 kg/m^2^ and had lost some degree of weight by the end of the study. However, due to the recruitment method, it was not possible to link the impact of the tool with actual weight changes. Only a few of the participants, those finding the tools helpful, cooked for themselves exclusively, while the majority cooked for themselves and somebody else. While household composition did not seem to be associated with experiencing barriers to the use of the tools, the eating context was frequently mentioned on occasions when the tools were not used (for example, going on holidays, during festivities, at somebody else´s home, or at work). This suggests that for some users, external control strategies (e.g., use of tableware) may not be sufficient and approaches targeting cognitive control may be necessary as an adjuvant therapy.

Our study builds on previous work looking at the effectiveness of portion-control tools [21,26,27] in a large sample of individuals attempting to manage their weight with minimal clinical contact. However, to our knowledge, this is the first fully qualitative analysis of public perceptions. Previous focus groups with the general public considered mostly two-dimensional aids, some of which have been discontinued or had low acceptability [14]. Other studies, examining the effect of serving utensils or plate size, did not include qualitative measures [23,37,38,39,40,41]. The only four existing studies examining portion-control tools for weight management did not explore the experiences of the participants in depth [21], or the intervention included dietetic counselling which makes it difficult to interpret the effect of the portion-control tool per se [15,26,27]. In a previous study involving sector plates and calibrated bowls, the investigators included instructions on how to use the tools for mixed meals (i.e., proportioning the food into the section that represented the dominant macronutrient of the meal) [21]. In retrospect, it would have been useful to include this information in our study as participants expressed difficulty or inconvenience in using the sector plate for mixed dishes. 

Participants consistently reported an educational benefit from the tools, perceiving that they acquired the ability to independently judge appropriate portions. This was important since consumption of large portion sizes of high energy density foods is a well-documented key contributor to obesity [42]. The educational benefits may be enhanced if these tools are used as part of a behavioral support program. Evidence from this study suggests it would be useful to educate users to use the tool correctly by avoiding loading it excessively or having extra helpings, on the importance of controlling portion size of energy-yielding beverages (for some individuals the calibrated glass was not perceived as necessary), and on healthy snacking (some participants increased snacking to compensate for smaller portions at mealtimes). Healthy snacking has been effectively incorporated into weight management regimes, whereas, unhealthy snacking can contribute substantially to the energy intake of individuals and lead to obesity [43]. More research is warranted on how to manage satiety, healthy snacking, and tailored portion sizes for those with higher energy requirements within a portion-control weight loss intervention. 

The exact mechanisms by which portion-control tools may affect energy intake are still not known. It is possible that in the current obesogenic environment, the process of selecting large portions has become an automatic behavior mediated by a combination of individual and environmental factors such as personal and social norms [44,45], unit bias [46], plate-cleaning tendencies [47], as well as the widespread availability of large portions, and financial incentives penalizing the consumption of smaller portions [7,9]. If portion size choice occurs beyond awareness [6,7] then interrupting this automatic process could in theory improve portion control. Portion tools may help this process by prompting users to pay additional attention to how much they self-serve of each meal component relative to the whole meal, mediated by the specific design of the tool and encouraging additional thought and planning [38,48]. In support of this point, participants consistently verbalized their surprise at how their habitual portions deviated from recommendations when using both tool sets. Other factors, such as increased satiety when eating a meal with the balanced proportions of each food group (i.e., vegetables, protein, and starch) defined by the tool, may also help, over time, to reset portion sizes [49]. Although the specific mechanisms of action cannot be determined from this study, these observations suggest that visual cues, memory, and expectations could play an important role in how portion-control tools work and these aspects are worthy of further investigation.

### 4.1. Limitations

The participant sample for this study was drawn from a group of patients with overweight and obesity, attending public weight loss services in the North of England, who were motivated to try the tools, and who may not be representative of the general population. The questionnaire was prepiloted amongst a sample of participants with obesity from the same geographical area and their feedback used to improve the design. Every effort was made to communicate that any answers were acceptable however the presence of others, or the fact that participants may be following a diet may have influenced the responses (for example, several of the respondents reported cooking for others in addition to themselves and about half of the participants reported following a healthy eating plan or a special diet). There was no wash-out period between experiencing the first and second tool set. However, there was no order effect in the quantitative indicators of ease of use and acceptance [22], which suggests that an order effect on the experiences of participants, if present, was probably small. Participants who responded to the open-ended questions were probably the ones who were more motivated to express their views, and the presence of some forced-choice questions may have primed respondents to raise these issues in the free text comment [50]. The intervention lasted only four weeks. The views and experiences of participants after using these tools for a longer period may be different. Despite this, a wide range of responses were obtained across the various open-ended questions suggesting that the most relevant themes were captured. While we obtained a richness of data from the questionnaire, structured interviews or focus groups with participants or the lifestyle advisors may have elicited more detailed information in the tool usage [51]. 

### 4.2. Implications for Practice

Overall the emergent themes suggested that the tools were positively received for portion control, probably mediated by their direct educational component. In addition, themes on usability, acceptability, and feasibility highlighted some barriers to the effectiveness of the tool through compliance with usage. In our parallel analysis, participants reported they would be fairly likely to continue using either of these tools if they were made available to them (on a scale 0–100, the crockery set was rated as 62% likely vs. 73% for the serving spoons) [22]. 

Currently, there is a growing awareness of the need for acceptability of the approaches used in health interventions. Here, we have identified a number of interrelated themes which could be used to inform interventions about facilitators and barriers to tool adherence. A tool that is educational and allows learning about appropriate portions has a higher potential for assisting with portion control and resulting in sustained behavior change than tools that do not induce learning. On the other hand, themes such as clarity, versatility, and usability that encompass unclear markings, divisions or instructions; finding it unsuitable for specific foods; diets or users; the tool allowing to “cheat”; and being inconvenient in transit, may act as barriers to use. These factors could create a negative experience and lead to a failure to achieve dietary goals, potentially decreasing long-term adherence to the tool. Tools must be a good “fit” in order to maximize the likelihood of adoption into routine eating practices. In line with this, suggested improvements by participants included expanding the instructions, making calibration marks clearer, and increasing the versatility of the tools for mixed meals, dry food, spreads, out of home use, and for other family members. Understanding the impact of such factors on adherence can inform future tool development. 

## 5. Conclusions

A strong educational benefit was a key emergent theme in this study with participants perceiving that they acquired the ability to independently judge portion sizes. The main benefit was perceived to be as a practical aid to learning about appropriate portion sizes for their needs (e.g., when trying to lose or maintain weight, but also for healthy eating), hence enabling them to achieve healthy food portioning behavior. Important barriers were also identified that impacted feasibility such as erratic use and compensatory eating. This suggests that while the overall experience for these participants was positive, a more supportive framework may be beneficial for some users. This may include individualized approaches such as strategies to manage snacking behavior, developing autonomy in food-related decisions (i.e., able to control themselves in piling up), and intuitive portion-control approaches (i.e., recognition of internal satiety signals). Further research may consider combining different sets of tools and exploring tools designed for children or for out-of-home eating, as well as a more in depth investigation of the mechanism of portion-control tools for influencing learning. 

## Figures and Tables

**Table 1:** Characteristics of participants (*n* = 29) in a qualitative portion-control tool study.

Characteristic	
Male, % (*n*)	37.9 (11)
Female, % (*n*)	62.1 (18)
Age, years (mean ± SD)	49 ± 13
White ethnic background, % (*n*)	96.6 (1)
History of mental illness, % (*n*)	10.3 (3)
BMI at baseline, kg/m^2^ (mean ± SD)	40.9 ± 5.8
Weight at baseline, kg (mean ± SD)	115.9 ± 22.5
Weight at end, kg (mean ± SD) ^1^	114.2 ± 21.5
Following special diet ^2^, % (*n*)	48.3 (14)
Previous experience with portion size tools, % (*n*)	3.4 (1)
Prepares meals for self only, % (*n*)	17.2 (5)
Prepares meals for self and others, % (*n*)	79.3 (23)

^1^ Maintained weight (*n* = 1), gained weight (*n* = 5), lost weight (*n* = 21). Two people did not provide a weight value at baseline. ^2^ Low carb diet (*n* = 5); low salt diet (*n* = 4); low fat diet (*n* = 2); other diet excluding kidney, liver and gluten-free diets (*n* = 3). SD, standard deviation.

**Table 2:** Results from the thematic framework analysis of responses to an open-ended questionnaire on the portion-control tool experience (*n* = 29). Overarching theme categories, themes and sub-themes are indicated, alongside examples of participant responses. The number of responses that fell into each category is indicated under each theme.

Over-Arching Themes	Themes	Sub-Themes (n of Participants Forming Sub-Theme)	Example of Response
Educational	Portion guidance on appropriate size Awareness of previously over-sized portions	Learning achieved (developed ability to control portions without tool) (*n* = 8) Confirmation of previous attempts to control through guesswork (*n* = 8) Feeling full on fewer calories (*n* = 2) Achieving portion goal (*n* = 10)	*The spoons helped me to realise how much bigger my original portion size was* (ID130, 51 years old, F, 34.4 kg/m^2^, lost 1.7 kg) *The fact that I now know my portions for some foods was too big* (ID116, 72 years old, M, 39.2 kg/m^2^, lost 1 kg) *Has been helpful but now using smaller plates for smaller portions* (ID109, 55 years old, M, 39.7 kg/m^2^, lost 4.2 kg)
Usability	Ease of use Functionality Versatility	Clarity and simplicity (*n* = 15) Helpful to measure portions accurately and precisely (*n* = 3) Remaining hungry (more snacking) (*n* = 3) Failure to achieve portion goal (*n* = 3) Easy to cheat with (snacking more, second helpings, piling upwards) (*n* = 5) Not suitable for certain foods (dry/raw, mixed meals) (*n* = 4) Not suitable for children, special diets (*n* = 2) Single person use (need plate for partner) (*n* = 1)	*Hassle-free and easy way to limit portions* (ID106, 31 years old, M, 54.4 kg/m^2^, lost 6.2 kg) *You could see on the plate the right amount of foods* (ID110, 47 years old, M, 44.9 kg/m^2^, gained 2.3 kg) *That the plate has made me stick to a small portion of meat and potatoes* (ID104, 33 years old, F, 30.8 kg/m^2^, lost 5.8 kg) [Liked aspect] *The amount that constitutes a portion* (ID140, 24 years old, F, 35.4 kg/m^2^, lost 2.4 kg) *It’s a bit messy to organize the food to sort it out* (ID117, 33 years old, M, 42.1 kg/m^2^, lost 10.5 kg) *It didn’t measure ‘mixed food’*(ID129, 37 years old, F, 35.9 kg/m^2^, lost 1.6 kg)
Acceptability	Quality Attractiveness of design	Weight (too light, too heavy) (*n* = 1) Material (too flimsy) (*n* = 8) Finish (visible glue, chipping easily) Size (too large, too small) (*n* = 9) Design (clever, easy to find, not blending in) (*n* = 9)	[Referring to crockery] *They look too small* (ID122, 39 years old, 34.7 kg/m^2^, M, lost 1.4 kg)
Feasibility	Convenience Appropriateness Adherence	Not convenient for eating on the go, at work Difficult during festivities, for eating with guests, for children (*n* = 5) Not seen as necessary (*n* = 3) Forgetting to use (*n* = 6)	*I would of liked to use the plate for my lunch. However taking it to work was inconvenient but I did use it to measure the portions* (ID140, 24 years old, F, 35.4 kg/m^2^, lost 2.4 kg)
Overall experience	Grateful for having tried/taking part Enthusiasm for tool Added value of tool Beneficial improvements	Wanting it for self and others (*n* = 8) Liking it but not planning to adhere (*n* = 4) Reducing waste (*n* = 4) Helping other family with portion control (*n* = 1) Easy to clean (*n* = 3) Saving time (*n* = 2)	*Wish I could buy a full set for everybody* (ID146, 37 years old, F, 54.7 kg/m^2^, lost 0.4 kg) *It helped considerably when portioning/serving food not only for myself but also my family, also helping to reduce less food wastage* (ID142, 57 years old, F, 44.3 kg/m^2^, missing data for weight change)

## Supplementary Materials

The following are available online at https://www.mdpi.com/2072-6643/11/5/1095/s1, File S1: Study questionnaire.
