# Supplementary material for: Exploring the Experiences of People with Obesity Using Portion Control Tools—A Qualitative Study
Source: Nutrients. 2019 May 17;11(5):1095. doi: 10.3390/nu11051095 (PMC6567225; doi:10.3390/nu11051095)
Supplement: Supplementary file 1 [file nutrients-11-01095-s001.pdf]

☐ CROCKERY SET
☐ SERVING SPOONS

Supplementary Material

Wirral Community NHS
NHS Trust

MRC Human Nutrition Research

Acceptance of portion size tools

Study Questionnaire

To the lifestyle advisor at the LWMS:

- Please fill in the box below before giving this questionnaire to the volunteer.
- Please give one copy of this questionnaire to each volunteer when they start the first portion tool trial (2 weeks) and a second copy when they start the second portion tool trial (2 weeks).
- Upon return, please fill in the date when each trial period was completed.

To be completed by Lifestyle advisor at the Lifestyle and Weight Management Centre, Wirral Community NHS Trust:

Volunteer ID number: ______________________

Date when this questionnaire was given out: ______________________

Study stage recently completed (please tick one only. Indicate last day of the 2 weeks trial period):

☐ First tool trial
Completed on: ………………….

☐ Second tool trial
Completed on: ……………………..

Volunteer instructions for completing this questionnaire

- The following questionnaire will aid the investigators of this study to learn more about your experience with the portion size tools you have tried.
- There are no right or wrong answers. Please be as honest as you can.
- The questionnaire consists of 10 questions, some of which have multiple parts. It should take you no more than 15 min to complete it.
- Once completed, **please return to your lifestyle advisor on your next visit.**

QUESTIONNAIRE

Dates when you used the tool: First day: .... Last day: ....

Question 1. Which tool have you just tried out? (please tick one):

☐ Crockery set ☐ Serving spoon set

Question 2. Over the last 2 weeks, how many days a week did you use this tool, or parts of it, on average?

☐ Never or once in the 2 weeks

☐ 1-2 days a week

☐ 3-5 days a week

☐ Most days of the week (6-7 days)

☐ Used it only for a few days at the beginning or at the end

Question 3. For which meals did you tend to use this tool (or parts of the tool)? (Tick all that apply)

☐ Breakfast ☐ Lunch ☐ Evening meal ☐ Snacks/drinks

Question 4. The following set of statements refers to your experience with *the specific tool or tool set* that you have just used (indicated in response to Question 1 above). Please indicate your level of agreement with each statement (please circle one number only):

	Strongly disagree				Strongly agree
a. I liked the look of the tool	**1**	**2**	**3**	**4**	**5**
b. This tool fits with the rest of my kitchenware	**1**	**2**	**3**	**4**	**5**
c. This tool fits with my family/home life	**1**	**2**	**3**	**4**	**5**
d. I felt embarrassed using this tool at home/ with others	**1**	**2**	**3**	**4**	**5**
e. This tool was easy to use	**1**	**2**	**3**	**4**	**5**
f. This tool was resistant to wear and tear	**1**	**2**	**3**	**4**	**5**
g. The written instructions provided with this tool were clear	**1**	**2**	**3**	**4**	**5**
h. Compared with other tools I have seen/tried, this tool was more convenient to use. (If you haven't used other tools before tick here ☐)	**1**	**2**	**3**	**4**	**5**

Question 4 (cont).

	Strongly disagree				Strongly Agree
i. This tool helped me learn what the appropriate portion size for particular foods is (e.g. for juice, pasta, or cereal)	**1**	**2**	**3**	**4**	**5**
j. I used the tool to measure foods I have never measured before	**1**	**2**	**3**	**4**	**5**
k. In general, once I used this tool for a particular food, I continued to use it for that food	**1**	**2**	**3**	**4**	**5**
l. Overall, this tool helped me meet my current dietary goals	**1**	**2**	**3**	**4**	**5**

Question 5. Was there any specific part or aspect of this tool that you found more useful? If so, please tell us which one(s) and why.

Question 6. We would like to ask you about how you used this tool. Please tell us if you think you have modified your usual portions when you have used the tool to prepare, serve or consume the following foods for yourself.

**Food**	**As a result of using the tool I have…** (Please tick one box only for each food group)
Vegetables (cooked)	☐ Increased size of serving (Increased)
☐ *Did not consume*	☐ Decreased size of serving (Decreased)
	☐ Have not changed the size of serving (Not changed)
	☐ Did not use tool for these foods
Salad, raw vegetables	☐ Increased
☐ *Did not consume*	☐ Decreased
	☐ Not changed
	☐ Did not use tool for these foods
Fruits (excluding fruit juice and smoothies)	☐ Increased
☐ *Did not consume*	☐ Decreased
	☐ Not changed
	☐ Did not use tool for fruits
Fruit juice, smoothies, fruit-flavoured drinks (such as in Cordial) and soft drinks	☐ Increased
	☐ Decreased
☐ *Did not consume*	☐ Not changed
	☐ Did not use tool for these drinks
Bread	☐ Increased
☐ *Did not consume*	☐ Decreased
	☐ Not changed
	☐ Did not use tool for bread
Rice and other grains	☐ Increased
☐ *Did not consume*	☐ Decreased
	☐ Not changed
	☐ Did not use tool for these foods
Breakfast cereals	☐ Increased
☐ *Did not consume*	☐ Decreased

- Not changed
- Did not use tool for breakfast cereals

Pasta<br>☐ *Did not consume*	☐ Increased<br>☐ Decreased<br>☐ Not changed<br>☐ Did not use tool for pasta
Potatoes (boiled or mashed)<br>☐ *Did not consume*	☐ Increased<br>☐ Decreased<br>☐ Not changed<br>☐ Did not use tool for this food
Chips, roast/wedge potatoes<br>☐ *Did not consume*	☐ Increased<br>☐ Decreased<br>☐ Not changed<br>☐ Did not use tool for these foods
Milk, yoghurt and milk-based drinks, including non-dairy alternatives<br>☐ *Did not consume*	☐ Increased<br>☐ Decreased<br>☐ Not changed<br>☐ Did not use tool for these foods
Cheese<br>☐ *Did not consume*	☐ Increased<br>☐ Decreased<br>☐ Not changed<br>☐ Did not use tool for cheese
Meat, fish and meat or fish-based dishes (e.g. meat or fish pies)<br>☐ *Did not consume*	☐ Increased<br>☐ Decreased<br>☐ Not changed<br>☐ Did not use tool for these foods
Pulses, egg-based dishes; meat-free foods (e.g. vegetarian options)<br>☐ *Did not consume*	☐ Increased<br>☐ Decreased<br>☐ Not changed<br>☐ Did not use tool for these foods
Savoury snacks (e.g. crisps; nuts)<br>☐ *Did not consume*	☐ Increased<br>☐ Decreased<br>☐ Not changed

	☐ Did not use tool for savoury snacks
Confectionary, cake, desserts<br>☐ *Did not consume*	☐ Increased<br>☐ Decreased<br>☐ Not changed<br>☐ Did not use tool for these foods
Butter; margarine<br>☐ *Did not consume*	☐ Increased<br>☐ Decreased<br>☐ Not changed<br>☐ Did not use tool for this food
Cooking oil, salad dressings<br>☐ *Did not consume*	☐ Increased<br>☐ Decreased<br>☐ Not changed<br>☐ Did not use tool for these foods
Alcoholic drinks<br>☐ *Did not consume*	☐ Increased<br>☐ Decreased<br>☐ Not changed<br>☐ Did not use tool for these drinks

Question 7. There might have been times when you think you could have used the tool provided but you didn't use it. Did this happen to you? If so, please tell us when this was and why (please note, there are no right or wrong answers here).

Question 8. Please indicate on the scale below *how likely it would be that you continue to use this tool* in the long-term (beyond 2 weeks) as part of your dietary plans, if the tool was available to you (please draw a vertical line on the appropriate point).

|———————————————————————|

Very unlikely — Extremely likely

Question 9. Please tell us what you liked and did not like about using this tool in the following situations.

a) When portioning or serving foods for yourself

I liked:<br><br><br>I did not like:

b) Using the tools for other purposes, e.g. servings others in your family; for recipe preparation; serving foods during social occasions, etc.

I liked:<br><br>I did not like:

☐ Tick here if you only used the tools for serving foods for yourself.

Question 10. In your opinion, what would help to make this tool(s) more acceptable and/or useful in helping you meet your current dietary goals?

Finally, you may use the space below for anything else you wish to tell us about your experience with this particular set of portion size tools.

This is the end of questionnaire.

THANK YOU VERY MUCH for your contribution.

Please return to your Lifestyle Advisor at your next visit.

If you have any questions regarding this questionnaire or would like further clarification on how to fill it in, please contact your Lifestyle Advisor or Dr. Eva Almiron-Roig at the Medical Research Council Human Nutrition Research Unit on:

Portion.tools@mrc-hnr.cam.ac.uk, tel. n. 01223 426356.
